# MiR‐26b suppresses hepatocellular carcinoma development by negatively regulating ZNRD1 and Wnt/β‐catenin signaling

**DOI:** 10.1002/cam4.2613

**Published:** 2019-10-21

**Authors:** Xiaobo Hu, Ruifang Wang, Zhigang Ren, Xiaorui Liu, Junsheng Gu, Guangying Cui, Qinggang Li

**Affiliations:** ^1^ Department of Infectious Diseases The First Affiliated Hospital of Zhengzhou University Zhengzhou China; ^2^ Department of Nuclear Medicine The First Affiliated Hospital of Zhengzhou University Zhengzhou China; ^3^ Gene Hospital of Henan Province Precision Medicine Center The First Affiliated Hospital of Zhengzhou University Zhengzhou China; ^4^ Henan Medical Key Laboratory of Molecular Imaging The First Affiliated Hospital of Zhengzhou University Zhengzhou China

**Keywords:** HCC, miR‐26b, Wnt/β‐catenin signaling pathway, ZNRD1

## Abstract

Previous studies have indicated that Zinc ribbon domain‐containing 1 (ZNRD1) is attributed to the carcinogenesis of human tumors. However, the role of ZNRD1 and its regulation in hepatocellular carcinoma (HCC) are still largely unclear. In this study, we examined the expression profiles of ZNRD1 in HCC tissues by immunohistochemistry (IHC) and publicly datasets analysis. In vitro and in vivo experiments were conducted to identify the function of ZNRD1 in HCC. In addition, miRNA potentially targeting ZNRD1 was predicted by bioinformatics analysis and further verified via in vitro experiments. Our results revealed that ZNRD1 was frequently upregulated in HCC tissues compared with that in nontumor tissues. High ZNRD1 expression in HCC tissues was positively associated with advanced tumor stage and poor prognosis. Function experiments showed that knockdown of ZNRD1 inhibited cell growth and invasion in vitro, and suppressed tumor development in vivo. Moreover, ZNRD1 promoted the activation of Wnt/β‐catenin signaling pathway in HCC. Importantly, miR‐26b directly inhibited the transcription activity of ZNRD1. Overexpression of ZNRD1 dramatically abolished the inhibitory effects of miR‐26b on HCC cells. Taken together, our results uncover a novel mechanistic role for miR‐26b/ZNRD1 axis in HCC, proposing ZNRD1 inhibition as a potent therapeutic strategy for hepatocellular carcinoma.

## Graphical abstract

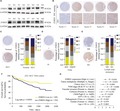

## 1 INTRODUCTION

Hepatocellular carcinoma (HCC) is a common digestive system malignancy that has high invasiveness and mortality.^1^, ^2^ Although the comprehensive therapeutic strategies have been employed, such as surgical resection, chemotherapy embolization and liver transplantation, a great amount of patients diagnosed at middle‐ or late‐stage HCC eventually have poor prognosis due to tumor recurrence and metastasis.^3^ Therefore, it is a pressing need to identity novel biomarkers and unravel the molecular mechanisms underlying HCC initiation and progression.

Zinc ribbon domain‐containing 1 (ZNRD1) was firstly identified as a Multi‐drug resistance (MDR)‐associated gene and overexpressed in drug‐resistant cancer cells.^4^ It has been reported that ZNRD1 depletion observably reversed drug resistance in gastric cancer and leukemia cells.^5^, ^6^, ^7^ Meanwhile, ZNRD1 could regulate the expression of P‐glycoprotein and BCL‐2.^7^ Enhanced expression of ZNRD1 was also observed in esophageal squamous cell carcinoma, and high level of ZNRD1 expression was statistically positively correlated with aggressive clinicopathologic features.^8^ However, the functional role of ZNRD1 in HCC development has not been fully explored yet.

MiRNAs are a group of small RNAs with ~22 nucleotides which play crucial roles in regulating gene expression mainly through binding to the 3′UTR region of target mRNAs.^9^ Emerging studies have revealed that dysregulation of miRNAs are involved in different kinds of malignancies developmental processes. Among them, MiR‐26b has been documented to be frequently downregulated in multiple cancers, such as gastric cancer, breast cancer, esophageal squamous cancer, prostate cancer, lung cancer, laryngeal cancer, and HCC.^10^, ^11^, ^12^, ^13^, ^14^, ^15^, ^16^, ^17^ On the contrary, the expression of miR‐26b is elevated in colorectal cancer, and miR‐26b overexpression could promote metastasis.^18^, ^19^ Therefore, the functional roles of miR‐26b in human tumors varied between different cancer types. Nevertheless, the function of miR‐26b in HCC progression and the molecular mechanisms through which miR‐26 influences the malignant phenotypes of HCC cells have not been fully understood.

In this study, we demonstrated that ZNRD1 was markedly overexpressed in clinical HCC tissues and positively correlated with poor prognosis. Knockdown of ZNRD1 attenuated cell proliferation and invasion ability in HCC cells in vitro. Moreover, ZNRD1 silencing had a profound inhibitory effect on HCC growth in vivo. We further demonstrated that ZNRD1 was a direct target of miR‐26b and restoration of ZNRD1 expression could reverse the miR‐26b‐mediated inhibition of the growth capacity of HCC cells through activating the Wnt/β‐catenin signaling pathway.

## 2 MATERIALS AND METHODS

### 2.1 Human specimens

HCC samples were collected between May 2014 and March 2016 at the First Affiliated Hospital of Zhengzhou University (Zhengzhou, China) as previously described.^20^ Tissue microarrays (TMA) containing 396 paired paraffin‐embedded HCC tissues (341 with available follow‐up data) and matched non‐neoplastic counterparts were obtained from patients with HCC who undergoing curative resection at the First Affiliated Hospital of Zhengzhou University as described previously. This study was approved by Institutional Review Board of the hospital.

### 2.2 Dataset acquisition and process

Expression data for ZNRD1 in HCC and additional cancer types were extracted from level 3 TCGA (The Cancer Genome Atlas) RNAseq data, and further validated by publicly available independent HCC microarray datasets (http://www.ncbi.nlm.nih.gov/geo/query/acc.cgi?acc=BLIRIJP, http://www.ncbi.nlm.nih.gov/geo/query/acc.cgi?acc=GSE36376, http://www.ncbi.nlm.nih.gov/geo/query/acc.cgi?acc=GSE39791, http://www.ncbi.nlm.nih.gov/geo/query/acc.cgi?acc=GSE45436, http://www.ncbi.nlm.nih.gov/geo/query/acc.cgi?acc=GSE54236, http://www.ncbi.nlm.nih.gov/geo/query/acc.cgi?acc=GSE54238, http://www.ncbi.nlm.nih.gov/geo/query/acc.cgi?acc=GSE57957, http://www.ncbi.nlm.nih.gov/geo/query/acc.cgi?acc=GSE64041, http://www.ncbi.nlm.nih.gov/geo/query/acc.cgi?acc=GSE76297, http://www.ncbi.nlm.nih.gov/geo/query/acc.cgi?acc=GSE76427, http://www.ncbi.nlm.nih.gov/geo/query/acc.cgi?acc=GSE77314, and http://www.ncbi.nlm.nih.gov/geo/query/acc.cgi?acc=GSE84005) from GEO (Gene Expression Omnibus) database as previously described.^21^ In addition, the relationship between the ZNRD1 expression and its clinical manifestations was validated by TCGA.

### 2.3 Cell culture and vector construction

Human HCC cell lines (Hep3B and SMMC‐7721) were stored in our laboratory. Cells were cultured in RPMI1640 or DMEM medium supplemented with 10% (FBS, Gibco), 100 U/mL penicillin sodium, and 100 mg/mL streptomycin sulfate at 37°C in a humidified air atmosphere with 5% CO_2_. ZNRD1 overexpression vector, shRNA vectors targeting ZNRD1 and negative control with scramble sequences were obtained from GenePharma and transfected into HCC cells using lipofectamine 3000 (Invitrogen) according to the manufacturer's protocol.

### 2.4 Immunohistochemistry (IHC)

Immunohistochemical staining was performed was performed according to previously described method.^22^ In brief, the TMA slides were deparaffinised through xylene, antigen retrieved and blocked with bovine serum albumin. Subsequently, the slides were incubated in ZNRD1 antibody diluted 1:200 at 4°C overnight and carried out using the EnVision™ system (DAKO) according to the manufacturer's instructions. The images of TMA slides were obtained using the NanoZoomer system (HP Inc), and analyzed by the software of the NDP.view 2.5.14 version.

### 2.5 Western blotting

Western blotting was performed according to previously described method.^23^ The antibodies used in this study were listed as following: ZNRD1 (sc‐393406, Santa Cruz), Wnt3a (sc‐136163, Santa Cruz), β‐catenin (51067‐2‐AP, Proteintech), APC (19782‐1‐AP, Proteintech), Cyclin D1 (26939‐1‐AP, Proteintech), GAPDH (60004‐AP, Proteintech).

### 2.6 Cell proliferation, invasion, and migration assay

CCK‐8 assay, cell migration, and invasion assay was performed according to previously described method.^23^

### 2.7 Dual‐luciferase reporter gene assay

Both WT and mutant 3′‐UTR of the ZNRD1 mRNA were cloned into the luciferase reporter vector (psiCHECK‐2, Promega). HEK293 cells were cotransfected with miR‐26b mimics and either ZNRD1‐wt or ZNRD1‐mut vectors. After 48 hours, the relative luciferase activity was analyzed using the Dual‐Luciferase Reporter Assay System (Promega).

### 2.8 HCC xenograft model

Mice experiments were approved by the Animal Health Committee of Zhengzhou University. The male nude mice (4‐6 weeks) were purchased from Beijing Vital River Laboratory. SMMC‐7721 cell lines transfected with ZNRD1 knockdown (sh‐ZNRD1) and empty lentivirus control (NC) were subcutaneously implanted into the lower flank of nude mice. Tumor growth was examined every week. Photographs were taken using the IVIS Lumina II system. The tumor tissues were weighed and extracted for further IHC staining.

### 2.9 Statistical analysis

All values were expressed as means ± standard deviation. The Student's *t* test, chi‐square test, univariate analysis, and multivariate analysis were used to evaluate the statistical significance. A value of *P* < .05 was considered to be statistically significant. All statistical analyses were performed with the SPSS 16.0 software (SPSS).

## 3 RESULTS

### 3.1 Upregulation of ZNRD1 in clinical HCC tissues is correlated with poor prognosis

**Figure 1:**
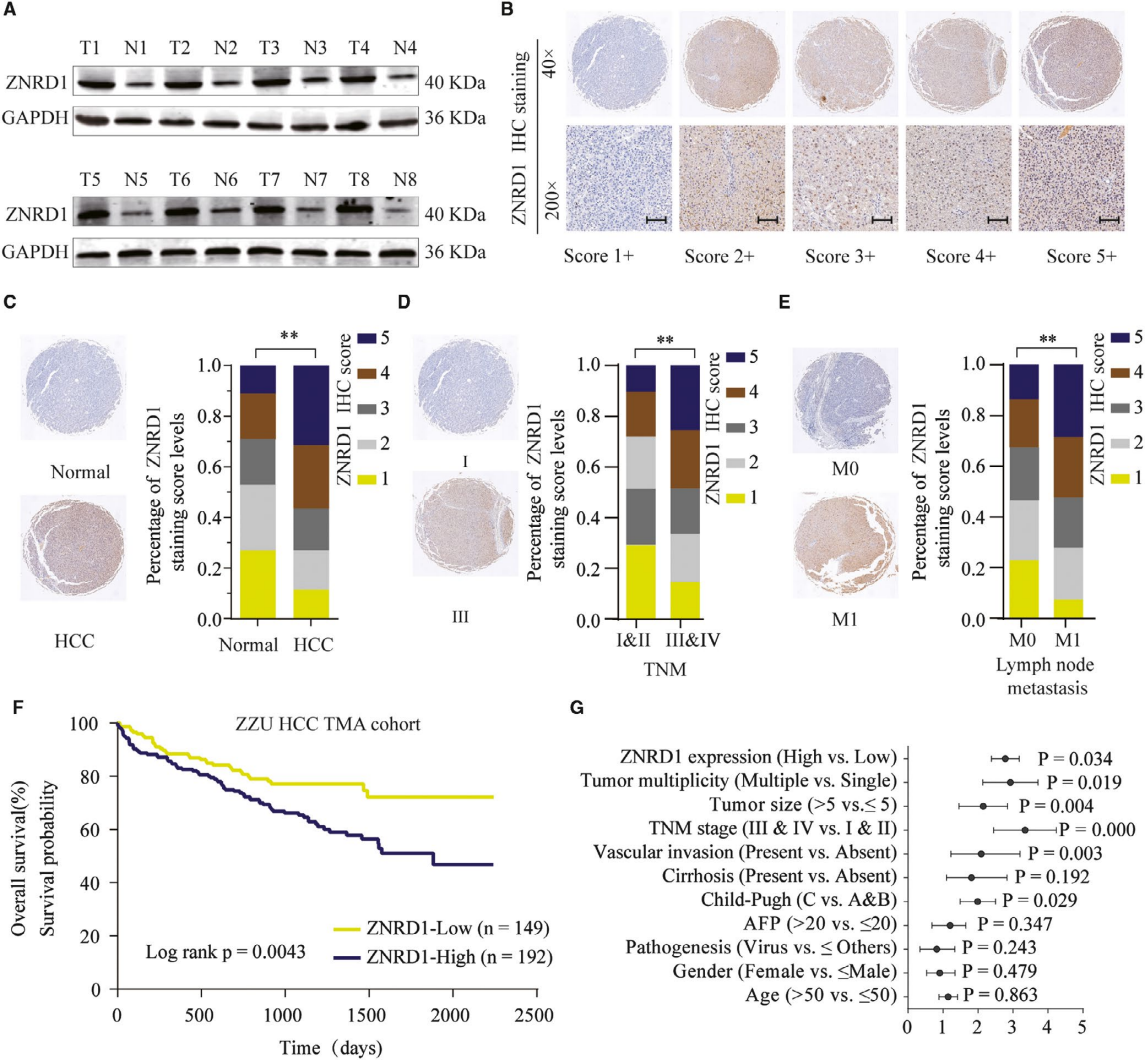
Zinc ribbon domain‐containing 1 (ZNRD1) is upregulated in hepatocellular carcinoma (HCC) tissues and predicts a poor prognosis. A, ZNRD1 expression in 8 paired HCC tissues and adjacent noncancer tissues was analyzed by western blot. B, Representative image of ZNRD1 IHC staining in HCC TMA cohort with different staining scores. Images were presented at 40× magnification (up panel) or 200× magnification (lower panel). C, Distribution of ZNRD1 IHC staining scores in HCC tissues or paired noncancer tissues. Images are presented at 40× magnification. D and E, Representative images of ZNRD1 IHC staining and distribution of ZNRD1 IHC staining scores in HCC tissues with different TNM stage, with or without lymph node metastasis. F, Kaplan‐Meier analysis of overall survival in patients with variable ZNRD1 expression. G, Univariate analysis of the association between ZNRD1 expression and different clinicopathological features. **P < .01 based on the nonparametric test

To identify the expression profile of ZNRD1 in HCC, ZNRD1 were examined by western blot in eight paired surgical HCC and surrounding nontumorous tissues. We found ZNRD1 was significantly increased in HCC tissues (Figure 1A). To further illuminate the relationship between ZNRD1 expression and HCC progression, we further analyzed ZNRD1 expression in TMA containing 396 paired HCC patients’ tissues. ZNRD1 IHC staining was classified into five subgroups according to IHC staining intensity (Figure 1B). Consistently, IHC staining further confirmed the significantly enhanced expression of ZNRD1 in HCC tissues (Figure 1C). Intriguingly, we found that ZNRD1 expression levels were positively correlated with HCC development stages (Figure 1D). Meanwhile, high expression levels of ZNRD1 in HCC were closely associated with lymph node metastasis (Figure 1E).

In addition, survival analysis showed that ZNRD1 high expression was positively associated with worse prognosis (Figure 1F). Moreover, results of univariate analysis indicated that high expression of ZNRD1 was correlated with late TNM stage, poor histological grade, and poor overall survival (OS) (Figure 1G; Table 1). In the multivariate analysis, ZNRD1 expression level was an independent prognostic factor for OS in HCC patients (Table 2). Therefore, these data suggest that high ZNRD1 expression is correlated with poor prognosis or survival in HCC.

**Table 1:** Correlation of clinicopathological features with ZNRD1 expression in ZZU HCC cohort

Clinicopathological variables features	ZNRD1 expression		*P*‐value
	Low expression (n = 149)	High expression (n = 192)	
Age (y)			
≤50	51 (34.2)	75 (39.0)	.359
>50	98 (65.8)	117 (61.0)	
Gender			
Male	120 (80.5)	145 (80.1)	.270
Female	29 (19.5)	47 (19.9)	
Pathogenesis			
Virus	110 (73.8)	145 (75.5)	.721
Others	39 (26.2)	47 (24.5)	
Cirrhosis			
Absent	135 (90.6)	180 (93.8)	.278
Present	14 (9.4)	12 (6.2)	
AFP			
≤20	90 (60.4)	76 (39.6)	**.000**
>20	59 (39.6)	116 (60.4)	
Portal vein thrombosis			
Absent	129 (86.6)	140 (72.9)	**.002**
Present	20 (13.4)	52 (27.1)	
TNM stage			
Stage I and II	126 (84.6)	137 (71.4)	**.004**
Stage III and IV	23 (15.4)	55 (28.6)	
Tumor size (cm)			
≤5	97 (65.1)	75 (39.1)	**.000**
>5	52 (34.9)	117 (60.9)	
Tumor multiplicity			
Single	84 (56.4)	75 (39.1)	**.002**
Multiple	65 (43.6)	117 (60.9)	
Survival state			
Live	121 (81.2)	109 (56.8)	**.000**
Dead	28 (18.8)	83 (43.2)	

Bold values indicate statistical significance, *P* < .05.

**Table 2:** Correlation of clinicopathological features with ZNRD1 expression in ZZU HCC cohort

	Univariate analysis			Multivariate analysis		
	HR	95% CI	*P* value	HR	95% CI	*P* value
Age (>50 vs ≤50)	1.143	0.884‐1.413	.863			
Gender (Female vs ≤ Male)	0.846	0.534‐1.342	.479			
Pathogenesis (Virus vs ≤Others)	0.784	0.341‐1.341	.243			
AFP (>20 vs ≤20)	1.270	0.684‐1.654	.347			
Child‐Pugh (C vs A&B)	1.814	1.491‐2.765	**.029**	1.468	1.271‐2.007	.247
Cirrhosis (Present vs Absent)	1.515	0.812‐2.825	.192			
Vascular invasion (Present vs Absent)	1.848	1.225‐2.793	**.003**	2.489	1.749‐3.015	**.022**
TNM stage (III & IV vs I & II)	3.333	2.262‐4.902	**.000**	4.158	3.029‐5.489	**.007**
Tumor size (>5 vs ≤5)	1.751	1.198‐2.564	**.004**	1.249	1.011‐1.698	.227
Tumor multiplicity (Multiple vs Single)	2.964	2.176‐3.472	**.019**	2.146	1.769‐2.465	**.037**
ZNRD1 expression (High vs Low)	2.765	2.102‐3.475	**.034**	3.125	2.498‐4.836	**.024**

Bold values indicate statistical significance, *P* < 0.05

### 3.2 Elevated ZNRD1 expression correlates with poor survival of HCC patients in public database

**Figure 2:**
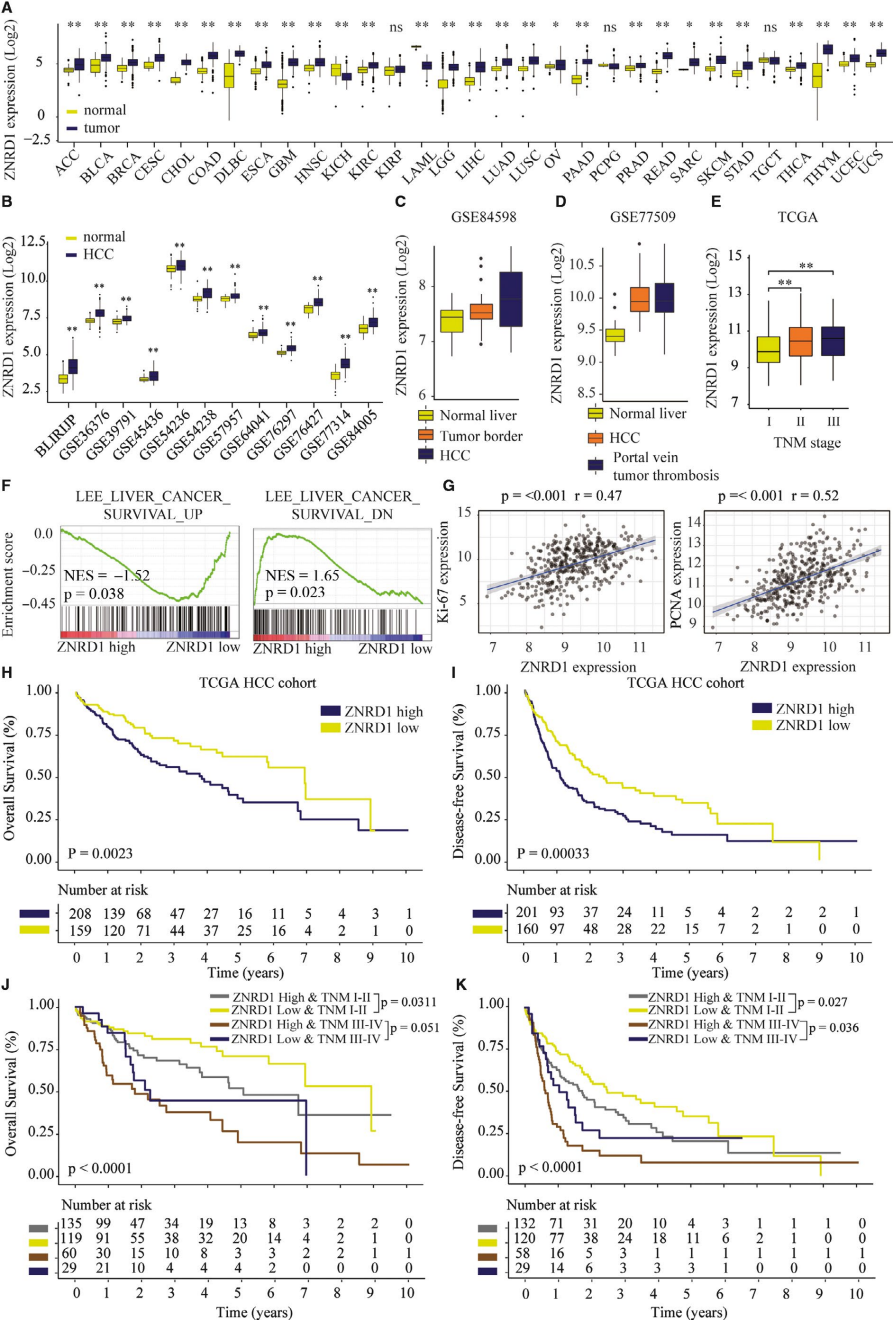
High Zinc ribbon domain‐containing 1 (ZNRD1) expression indicates poor prognosis in hepatocellular carcinoma (HCC) patients from TCGA cohort. A, ZNRD1 mRNA expression was analyzed in various cancers based on the dataset from TCGA and GTEX pancancer database. B, Bioinformatics analysis of ZNRD1 expression in HCC or nontumor tissues based on the dataset from TCGA and GEO database. C, Bioinformatics analysis of ZNRDF1 expression in normal liver, tumor border, and HCC in http://www.ncbi.nlm.nih.gov/geo/query/acc.cgi?acc=GSE84598 dataset. D, Bioinformatics analysis of ZNRD1 expression in normal liver, HCC, and portal vein tumor thrombosis in http://www.ncbi.nlm.nih.gov/geo/query/acc.cgi?acc=GSE77509 dataset. E, Bioinformatics analysis of ZNRD1 expression in HCC tissues with different TNM stages based on the dataset from TCGA. F, The Gene Set Enrichment Analysis of the correlation between ZNRD1 expression and gene signatures of survival in HCC. G, The Pearson correlation of ZNRD1 levels with Ki‐67 and PCNA expression in TCGA‐HCC cohort. Kaplan‐Meier analysis of the correlation between ZNRD1 expression and overall survival (OS) rate (H) or disease‐free survival (DFS) (I) in TCGA HCC cohorts. J and K, Kaplan‐Meier analysis of the correlation between ZNRD1 expression and OS, DFS of patient at different TMN stages in TCGA HCC cohorts. *P < .05, **P < .01 based on the nonparametric test

To further evaluate the expression pattern and prognostic role of ZNRD1 in human HCC, we analyzed the datasets from public available cancer microarray database (GTEX and TCGA). ZNRD1 mRNA levels were frequently upregulated in most cancer types, including HCC (Figure 2A). To further testify the analytical result, 16 HCC microarray datasets were also collected and analyzed. The results showed the remarkable elevated ZNRD1 expression in 12 of 16 datasets (Figure 2B). Moreover, data form http://www.ncbi.nlm.nih.gov/geo/query/acc.cgi?acc=GSE77509 and http://www.ncbi.nlm.nih.gov/geo/query/acc.cgi?acc=GSE84598 showed that ZNRD1 expression was markedly higher in HCC tissues than that in portal vein tumor thrombosis (Figure 2C) and tumor border (Figure 2D). Higher ZNRD1 expression was significantly associated with advanced TNM stages (Figure 2E).

Gene set enrichment analysis (GSEA) of the TCGA HCC dataset revealed that ZNRD1 expression was significantly associated with gene signatures of cell survival (Figure 2F). Furthermore, we found that the expression of ZNRD1 was positively correlated with the expression of proliferation markers Ki67 and PCNA (Figure 2G). Kaplan‐Meier curve analysis showed that patients with higher ZNRD1 expression had poor OS rate and disease‐free survival (DFS) rate (Figure 2H,I). Subgroup analysis showed that HCC patients with high ZNDR1 expression had significant lower OS and DFS than those with low ZNRD1 expression both in early (stages I and II) and late‐stage (stages III and IV) patients (Figure 2J,K), indicating that upregulated ZNDR1 might be a potential prognostic biomarker besides TNM stage.

### 3.3 Silencing ZNRD1 inhibits HCC cell proliferation, migration, and invasion in vitro

**Figure 3:**
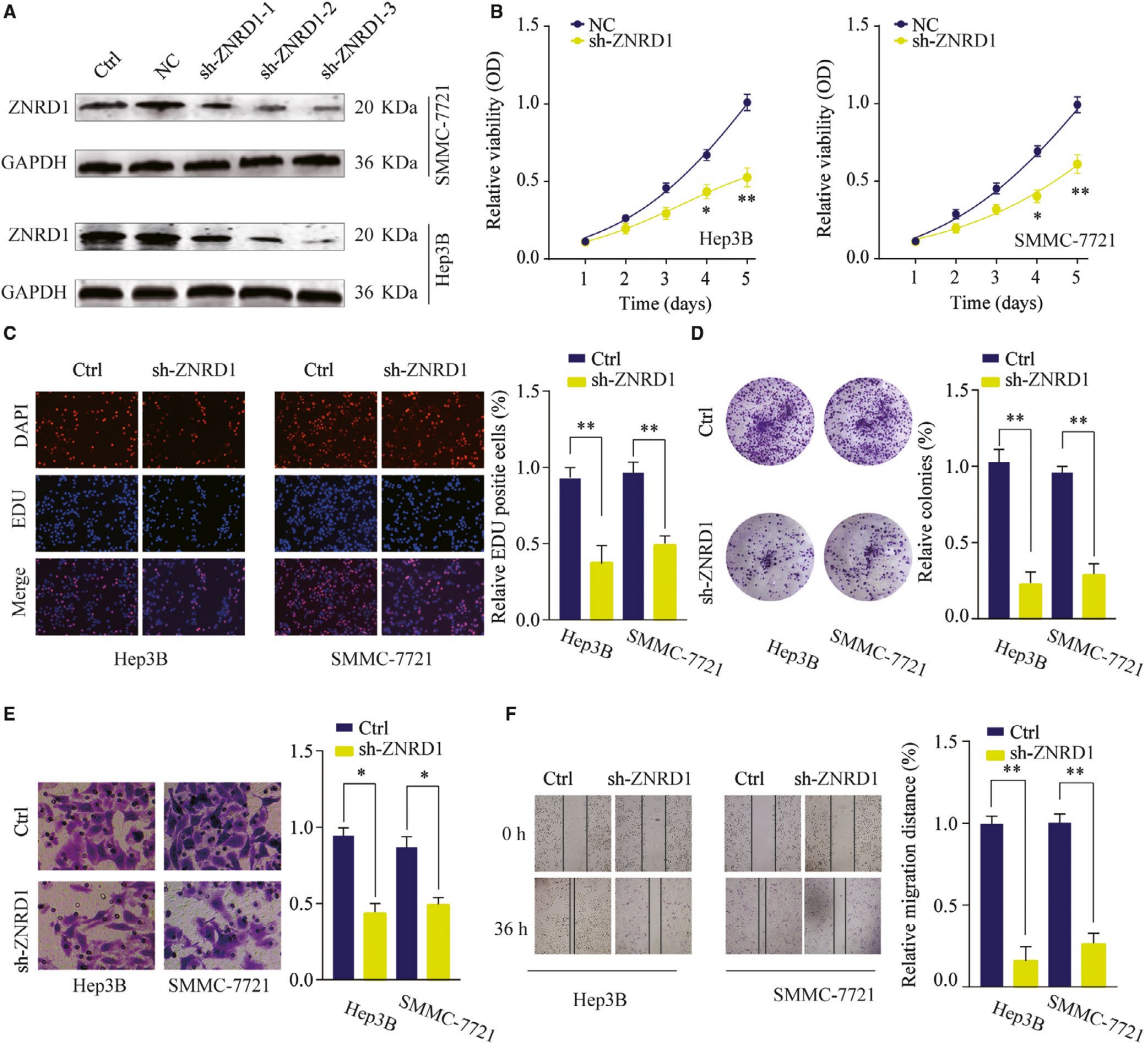
Zinc ribbon domain‐containing 1 (ZNRD1) silencing inhibits hepatocellular carcinoma (HCC) cell proliferation, migration, and invasion. A, HCC cells SMMC‐7721 or Hep 3B were transfected with negative control (NC) or different shRNAs targeting ZNRD1. The knockdown efficiency was analyzed by western blotting 48 h later. GAPDH was used as a loading control. B, Effects of ZNRD1 knockdown on cell proliferation were determined by CCK‐8 cell proliferation assays. Data were represented as the means ± SD. C, DNA synthesis in HCC cells SMMC‐7721 or Hep 3B was measured using the EdU incorporation assay. D, Effects of ZNRD1 knockdown on cell proliferation were determined by colony formation assay. E, Transwell assays were performed to evaluate the effect of ZNRD1 knockdown on cell invasion capability. Cells were counted under a microscope in five randomly selected fields. F, Wound‐healing assays were performed to evaluate the effect of ZNRD1 knockdown on cell migration. *P < .05, **P < .01

To further study the function of ZNRD1 in HCC, we transfected shRNAs targeting ZNRD1 into Hep3B and SMMC‐7721 cells to knockdown ZNRD1 expression (Figure 3A). The biological function of ZNRD1 was evaluated using the most efficient sh‐ZNRD1. CCK‐8, EdU staining, and colony‐forming assays showed that ZNRD1 knockdown significantly inhibited cell proliferation, DNA synthesis, and colony formation ability of Hep 3B and SMMC7721 cells (Figure 3B‐D). Furthermore, transwell and wound‐healing assays demonstrated that ZNRD1 knockdown led to decreased cell invasion and migration ability of Hep3B and SMMC7721 cells (Figure 3E,F). In summary, the results suggest that ZNRD1 might function as an oncogene and promote HCC cell proliferation, migration, and invasion.

### 3.4 Knockdown of ZNRD1 suppresses HCC tumor growth in vivo

**Figure 4:**
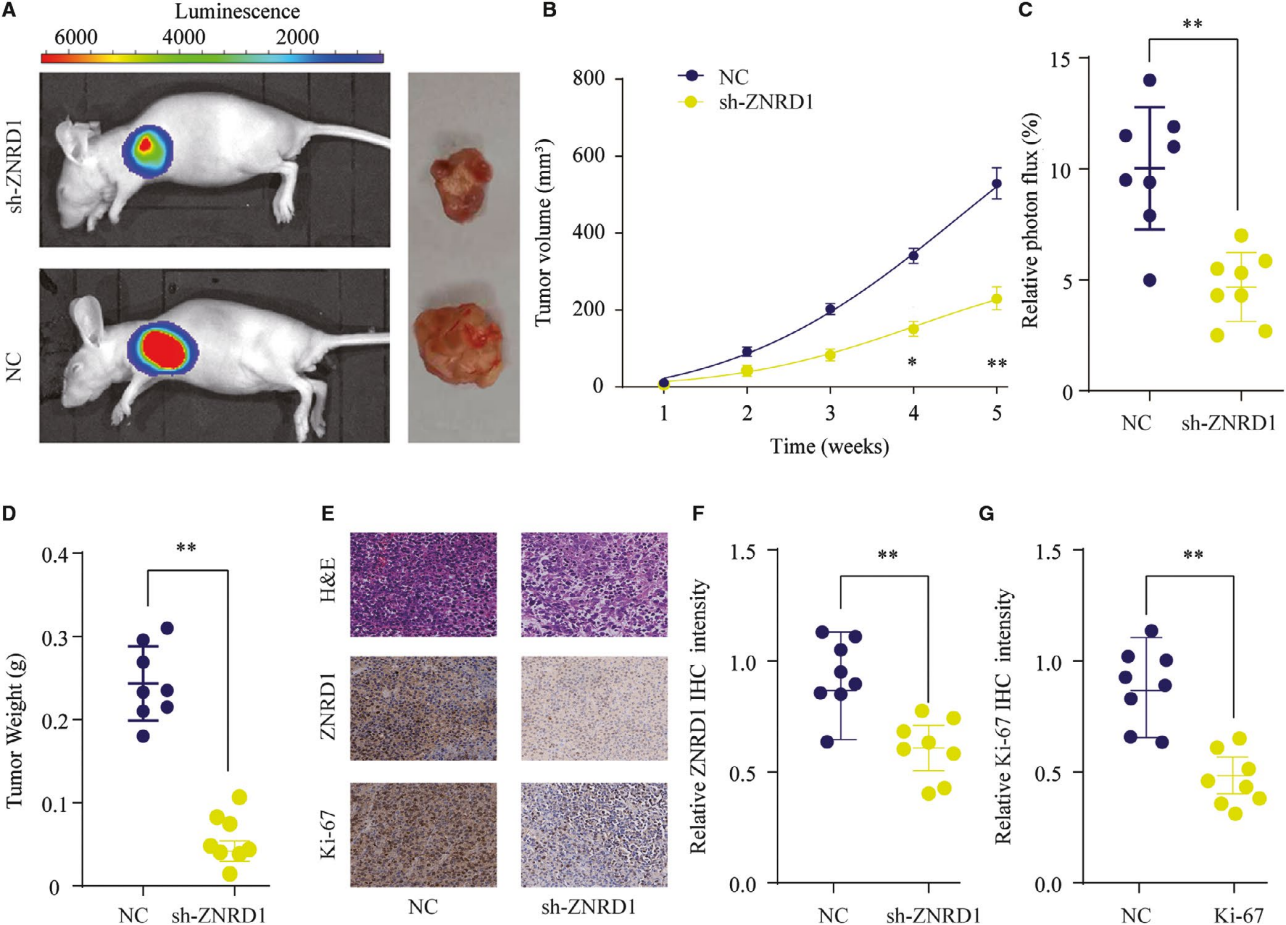
Zinc ribbon domain‐containing 1 (ZNRD1) silencing suppresses tumorigenesis in nude mice. Hepatocellular carcinoma SMMC‐7721 cells transfected with Ctrl or sh‐ZNRD1 were implanted subcutaneously into nude mice and tumor growth was monitored. A, Representative photos of nude mice and colon tumor tissues from Ctrl or sh‐ZNRD1 group at week 5. B, Growth curves of subcutaneous xenografts were determined based on tumor size measured every week. C, Relative photon flux of tumors in Ctrl or sh‐ZNRD1 group was determined by quantifying the bioluminescent signal. D, Tumor weight were measured and represented as means of tumor weights ± SD. E, The representative images of H&E staining, ZNRD1 and Ki‐67 IHC staining of tumor sections from Ctrl or sh‐ZNRD1 group. F and G, Quantification of relative ZNRD1 and Ki‐67 staining intensity in tumor sections from the Ctrl or sh‐ZNRD1 group. *P < .05, **P < .01

We also evaluated the function of ZNRD1 in vivo using HCC xenograft tumor model. As shown in Figure 4A, mice inoculated with Hep 3B cells knocking down ZNRD1 (sh‐ZNRD1) had markedly delayed tumorigenesis and smaller size of tumors compared with that of control group. In addition, tumors from sh‐ZNRD1 group had significantly lower weight in comparison with those from sh‐NC group (Figure 4B‐D). Moreover, IHC staining showed that the tumors from sh‐ZNRD1 group displayed significantly reduced the expression of ZNRD1 and Ki‐67 compared with that in tumors from sh‐NC group (Figure 4E‐G). These results suggest that suppressing ZNRD1 expression in HCC cells could efficiently inhibit tumorigenesis in vivo.

### 3.5 ZNRD1 activates the Wnt/β‐catenin signaling pathway

**Figure 5:**
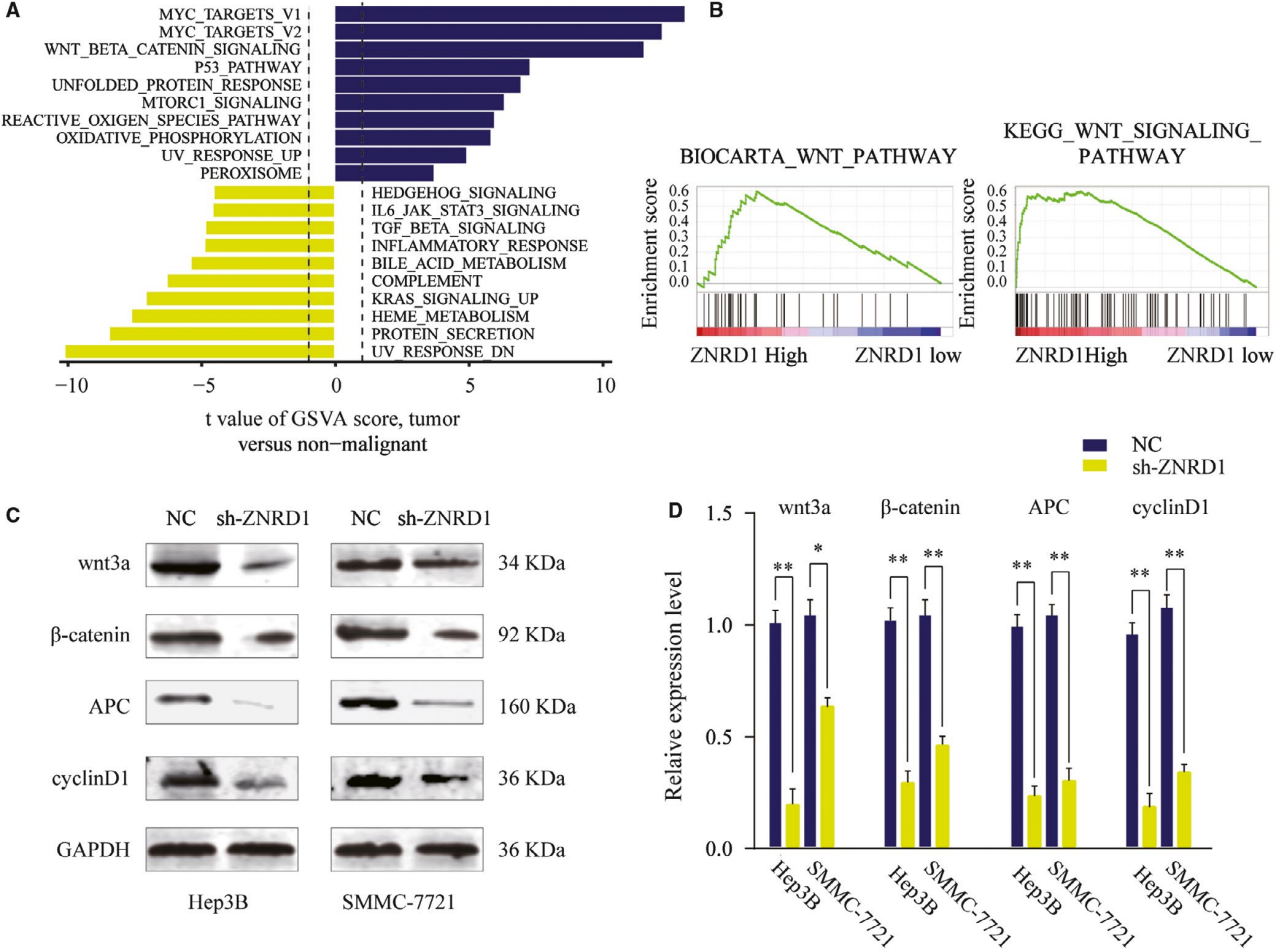
Zinc ribbon domain‐containing 1 (ZNRD1) activates Wnt/β‐catenin signaling pathway in hepatocellular carcinoma (HCC). A, GSVA analysis showed that Wnt/β‐catenin pathway in tumor compared with adjacent nontumor were identified as the top three activated regulated pathway in TCGA HCC cancer cohort. B, The Gene Set Enrichment Analysis of the correlation between ZNRD1 expression and gene signatures of Wnt/β‐catenin signaling pathway in HCC. C and D, SMMC‐7721 or Hep 3B cells were transfected with Ctrl or sh‐CLK3 and the expression of Wnt3a, β‐catenin, APC, and cyclinD1 was analyzed by western blot. Relative quantification analysis was based on grayscale values. *P < .05, **P < .01

We then searched for the potential mechanism of the tumor promotion induced by ZNRD1 in HCC cells using comprehensive bioinformatics analysis in TCGA HCC cohort. Gene Set Variation Analysis (GSVA) was conducted to predict the potential signaling pathways involved. As shown in Figure 5A, GSVA results indicated that ZNRD1 might be involved in the regulation of Wnt/β‐catenin signaling pathway. Consistently, GSEA results further confirmed that there was a positive correlation between ZNRD1 expression and Wnt/β‐catenin signaling pathway (Figure 5B). Western blot assays were performed and the results showed that the expression levels of wnt‐3a, β‐catenin, APC, and cyclin D1 were significantly decreased when ZNRD1 was silenced in Hep3B and SMMC7721 cells (Figure 5C,D). Taken together, these findings suggest that ZNRD1 might regulate the activation of Wnt/β‐catenin signaling pathway.

### 3.6 ZNRD1 is a direct functional target of miR‐26b

**Figure 6:**
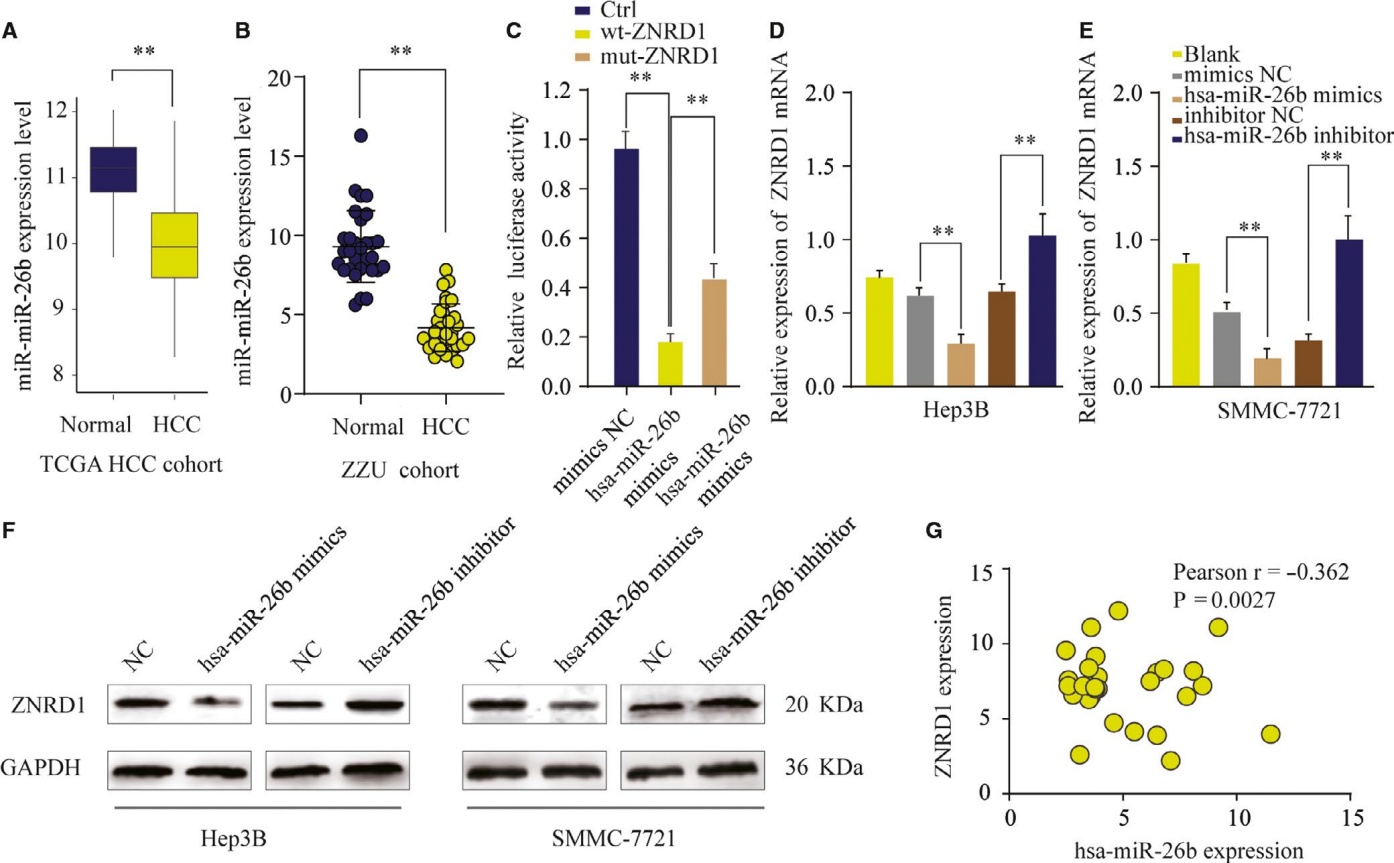
Zinc ribbon domain‐containing 1 (ZNRD1) is a direct target of miR‐26b in hepatocellular carcinoma (HCC) cells. A and B, The expression level of miR‐26b in HCC and nontumor normal tissues was analyzed in TCGA and ZZU cohort. C, The relative luciferase activities were detected in 293T cells transfected with ZNRD1 wild‐type and mutant type plasmids based on a dual‐luciferase reporter assay. D‐F, The expression levels of ZNRD1 mRNA (D, E) or protein (F) in SMMC‐7721 or Hep 3B cells transfected with miR‐26b mimic, miR‐26b inhibitor or negative ctrl were analyzed by qRT‐PCR or western blot. G, Pearson correlation analysis of the miR‐26b expression and ZNRD1 expression in HCC tissues. **P < .01

Studies have reported that ZNRD1 could be regulated by miRNAs in other cancer.^24^ Thus, we performed comprehensive bioinformatics analysis using online database TargetScan. miR‐26b was predicted to have the putative complementary binding sites on 3’‐UTR of ZNRD1. We further examined the expression pattern of miR‐26b and found that the expression of miR‐26b was significantly decreased in HCC tissues compared with that in paired normal tissues in TCGA HCC cohort (Figure 6A). The lower the expression of miR‐26b was further confirmed in our own clinical HCC tissues from ZZU cohort (Figure 6B). Luciferase reporter assay showed that miR‐26b mimics specifically and potently reduced the luciferase activity in HEK293 cells transfected with reporter vector containing WT 3’‐UTR of ZNRD1, but not the reporter vector containing mutated 3’‐UTR of ZNRD1 (Figure 6C). To further analyze the regulation of ZNRD1 by miR‐26B, we treated Hep3B or SMMC7721 cells with miR‐26b mimics or miR‐26b inhibitor. We found that miR‐133b mimics significantly reduced the ZNRD1 expression, whereas miR‐26b inhibitor enhanced ZNRD1 expression in mRNA level (Figure 6D,E), and similar result were observed in protein level through western blot analysis (Figure 6F). Moreover, Pearson association analysis showed a negative correlation between miR‐26b expression and ZNRD1 expression (Figure 6G). Collectively, these data suggest that ZNRD1 is a direct target of miR‐26b in HCC.

### 3.7 MiR‐26b exerts its function by suppressing ZNRD1 expression in HCC cells

**Figure 7:**
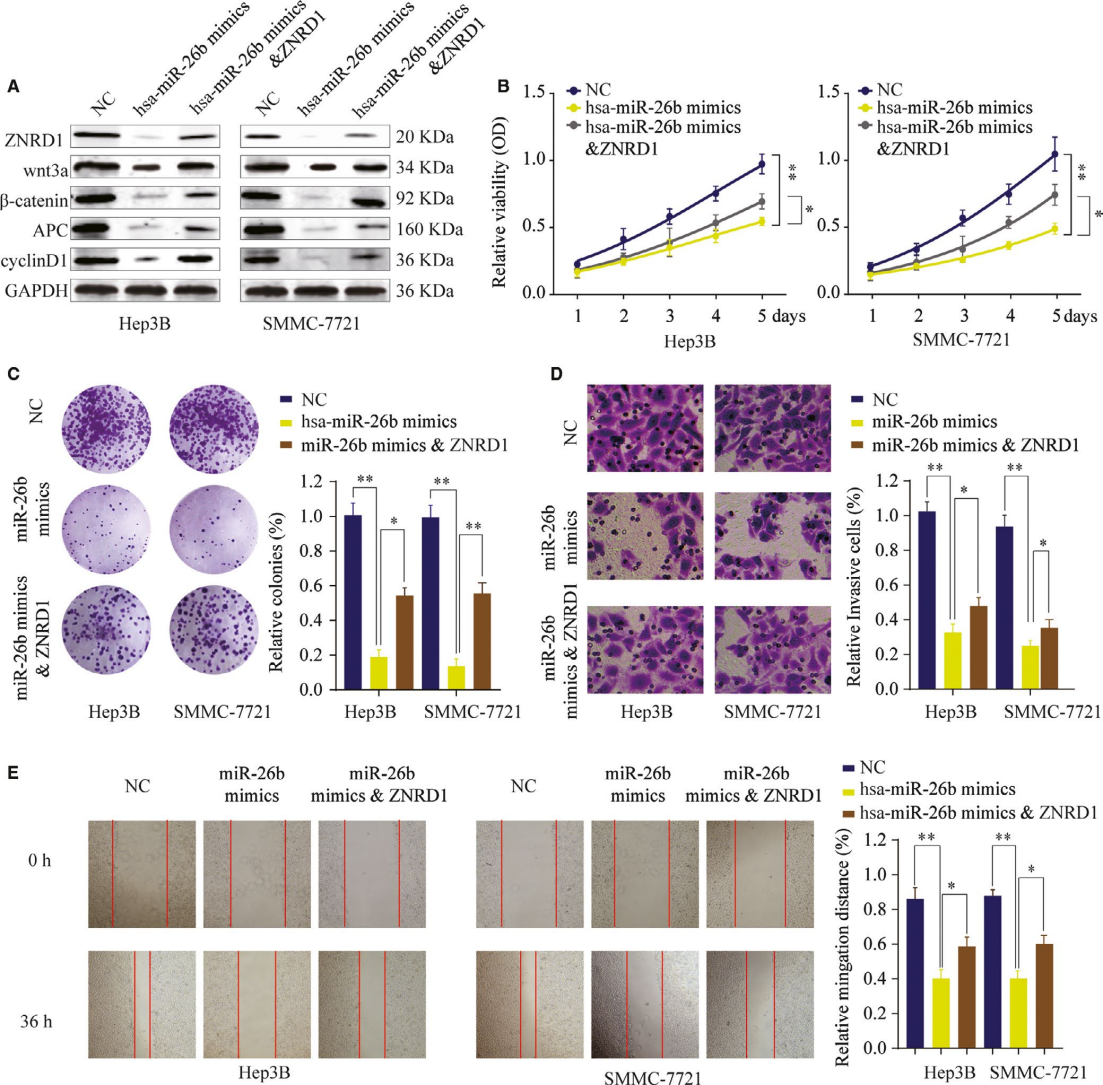
MiR‐26b inhibits hepatocellular carcinoma cell proliferation, invasion and migration in vitro by targeting Zinc ribbon domain‐containing 1 (ZNRD1). SMMC‐7721 or Hep 3B cells were transfected with NC, miR‐26b mimics or miR‐26b mimics & ZNRD1 overexpression plasmid. A, The expression levels of ZNRD1, Wnt3a, β‐catenin, APC, and cyclinD1 were analyzed by western blot 48 h later. B, Cell proliferation was determined by CCK‐8 cell proliferation assays. C, Colonies of SMMC‐7721 or Hep 3B in different groups were determined by colony formation assay. D, Cell invasion capability of SMMC‐7721 or Hep 3B in different groups was analyzed by transwell assay. E, Cell migration capability of SMMC‐7721 or Hep 3B cells in different groups was analyzed by wound‐healing assay. *P < .05, **P < .01

To investigate whether miR‐26b exerts its function via regulating ZNRD1, Hep3B, or SMMC7721 cells transfected with negative control, miR‐26b mimics, or miR‐26b mimics together with ZNRD1 overexpression plasmid. As shown in Figure 7A, miR‐26b suppressed the expression of ZNRD1, however, ZNRD1 expression was restored in by ZNRD1 overexpression in miR‐26b mimics + ZNRD1 group in cotransfected HCC cells. Meanwhile, ZNRD1 overexpression could partially reverse the miR‐26b induced inhibition of Wnt/β‐catenin signaling pathway (Figure 7A). Function experiments also showed that cell growth inhibition induced by miR‐26b overexpression was relieved by transfection with ZNRD1 plasmid (Figure 7B,C). In addition, wound‐healing and transwell assays demonstrated that the effect of miR‐26b inhibition on cell migration and invasion was abolished by ZNRD1 overexpression (Figure 7D,E). These results indicate that miR‐26b might serve as a tumor suppressor by inhibiting the proliferation, migration, and invasion of HCC cells through, at least in part, targeting ZNRD1.

## 4 DISCUSSION

In this study, we investigated the function of ZNRD1 in HCC, as well as the potential underlying mechanisms. First, we found that ZNRD1, as a novel oncogene in HCC, was significantly upregulated in HCC tissues. Moreover, ZNRD1 expression levels were positively correlated with the clinical tumor stage and lymph node metastasis. Meanwhile, our results indicated that high expression of ZNRD1 might be closely associated with poor prognosis in HCC to the best of our knowledge, this is the first report on the expression profiling and prognostic value of ZNRD1 in HCC. These findings demonstrate that ZNRD1 may play a tumor‐promoting role in HCC and prompt us to study its biological function.

To better understand the function of ZNRD1 in HCC, we performed functional assay to evaluate the biological function of ZNRD1 both in vitro and in vivo. We showed that ZNRD1 silencing inhibited the proliferation, colony formation, migration, and invasion capacity of HCC cells. In accordance with our results, Hong et al found that gastric cancer cells proliferation was dramatically inhibited after knocking down ZNRD1.^25^ We also confirmed the oncogenic role of ZNRD1 in vivo using xenograft model and showed knockdown of ZNRD1 remarkably inhibited HCC tumor development. Thus, these results indicate that ZNRD1 plays a crucial role in the progress and metastasis of HCC.

To determine that whether or not miRNAs were involved in the regulation of ZNRD1 expression, we employed an integrated approach using public bioinformatics tools. ZNRD1 was predicted as a direct target of miR‐26b and the interaction between miR‐26b and ZNRD1 was validated via luciferase report assay. Moreover, functional assays confirmed that ZNRD1 was a target of miR‐26b and ZNRD1 overexpression could partially reverse the inhibitory function of miR‐26b on cell proliferation, migration, and invasion. Studies demonstrated that miR‐26b could regulate HCC epithelial‐mesenchymal transition and metastasis through targeting USP9X and SMAD1.^26^, ^27^ Li et al reported that miR‐26b could inhibit HCC cell invasion and migration by directly binding to gene EphA2.^28^ In addition, miR‐26b was reported to enhance the chemosensitivity of HCC cells by targeting TAK1.^13^ Thus, these findings suggest that miR‐26b plays a crucial role in HCC as a tumor‐suppressing miRNA.

To understand the underlying signaling pathway involved in ZNRD1 regulated HCC progression, bioinformatics, and mechanism studies were conducted and results indicated that Wnt/β‐catenin signaling‐related targets were upregulated in HCC cells transfected with sh‐ZNRD1. Emerging evidence has indicated that constitutive activation of Wnt/β‐catenin signaling was a major hallmark in various cancers, including HCC.^29^ Our findings indicate that the miR‐26b/ZNRD1 axis contributes to progression of HCC through regulating the Wnt/β‐catenin signaling. Previous studies reported that ZNRD1 was implicated in the development of cancers multidrug resistance. Hong et al reported that ZNRD1 mediated multidrug resistance of gastric cancer and leukemia cells.^7^, ^30^ However, whether ZNRD1 is involved in HCC drug resistance remains unknown and future work will be dedicated to address these issues.

In conclusion, we demonstrated that ZNRD1 was upregulated in HCC cell lines and tissues. Knockdown of ZNRD1 inhibited HCC proliferation and invasion both in vitro and in vivo by regulating Wnt/β‐catenin signaling. Furthermore, we identified ZNRD1 was a direct target of miR‐26b and miR‐26b could inhibit HCC cell proliferation and invasion via regulating ZNRD1. In summary, our results suggest a profound insight into the development of HCC, and provide a potential therapeutic target for the treatment of HCC.

## CONFLICT OF INTEREST

The authors report no conflicts of interest in this work.

## AUTHOR CONTRIBUTIONS

HXB and LQG designed the study. HXB, WRF, RZG, and LXR performed the experiments. GJS and CGY analyzed the data. HXB and LQG wrote the manuscript. All authors reviewed and approved the manuscript.
